# Supplementary material for: Gastrointestinal Tract Stabilized Protein Delivery Using Disulfide Thermostable Exoshell System
Source: Int J Mol Sci. 2022 Aug 30;23(17):9856. doi: 10.3390/ijms23179856 (PMC9456531; doi:10.3390/ijms23179856)
Supplement: Supplementary file 1 [file ijms-23-09856-s001.zip › ijms-1830506-supplementary.pdf]

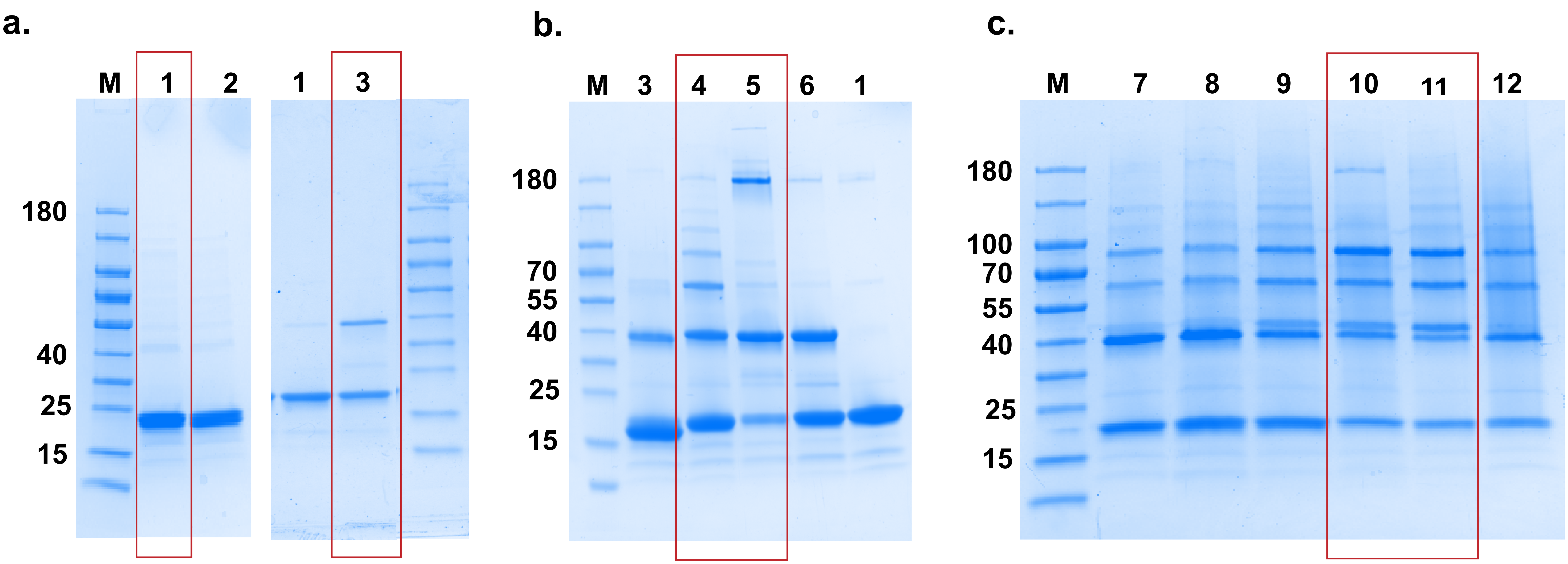

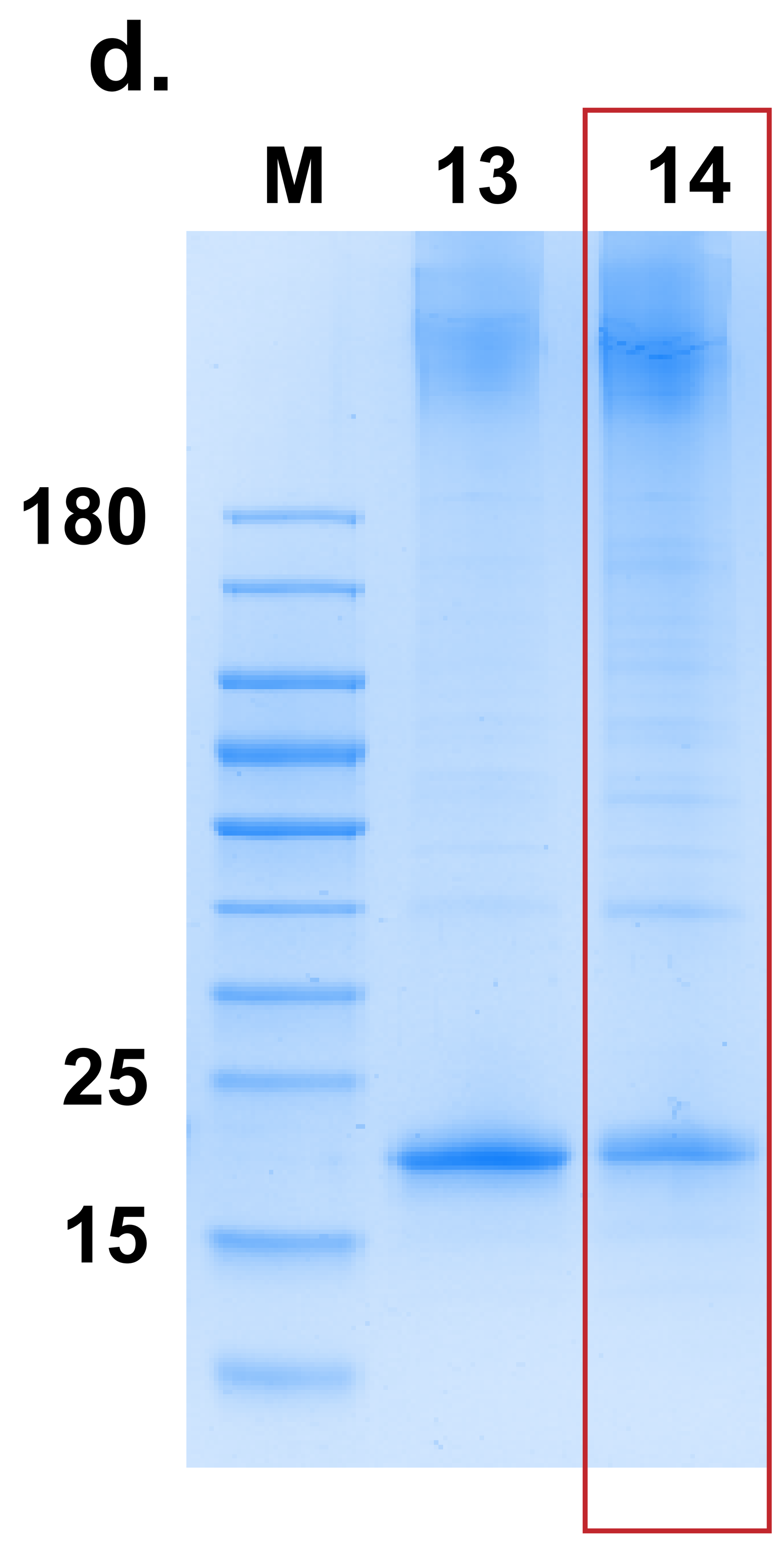

1. tES
2. tES R151C
3. tES A152C
4. tES A152C - L53C
5. tES A152C - A74C
6. tES A152C - G76C
7. tES A152C - R66C
M. Marker
8. tES A152C - L53C - R66C
9. tES A152C - A74C - R66C
10. tES A152C - G67C
11. tES A152C - L53C - G67C
12. tES A152C - A74C - G67C
13. tES A152C - G67C - A117C - G37C
14. tES A152C - L53C - G67C - A117C - G37C

Figure S1. (a-d) Non-reducing SDS-PAGE for designed cysteine residue substitutions.

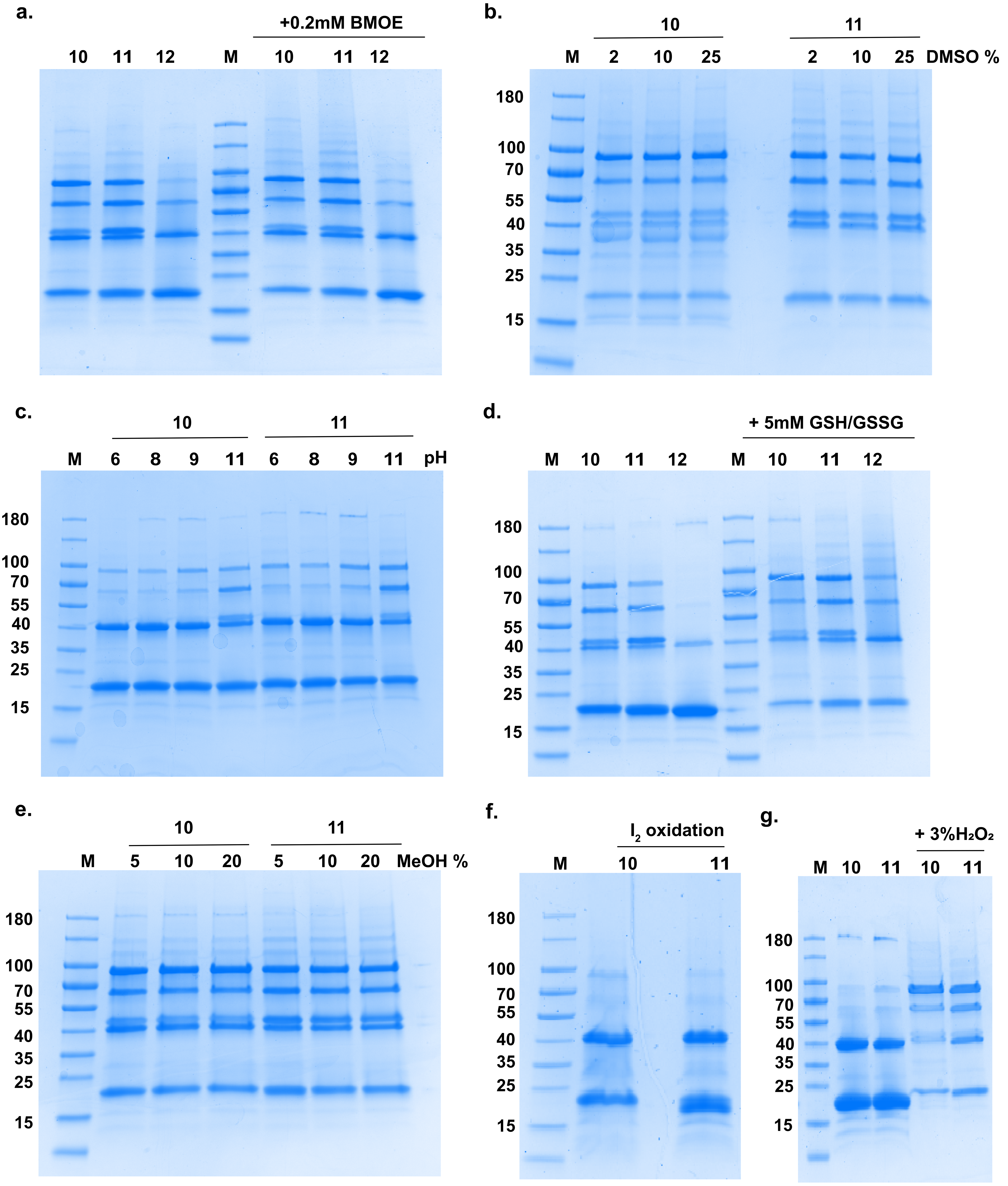

9. tES A152C - A74C - R66C
10. tES A152C - G67C
11. tES A152C - L53C - G67C
M. Marker

Figure S2. (a-g) Non-reducing SDS-PAGE to increase disulfide formation in 9,10, and 11 constructs.

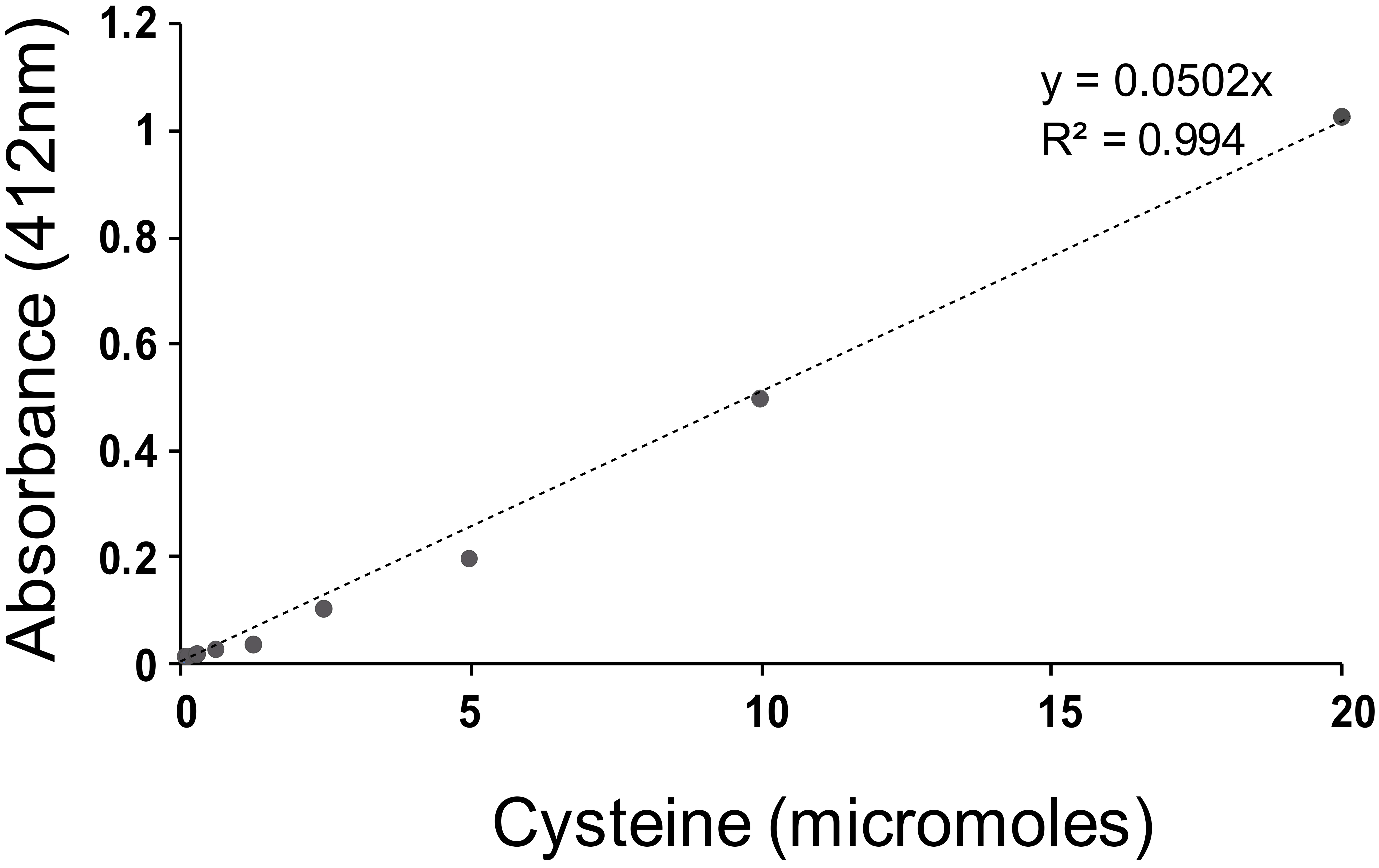

Figure S3. Ellman's Assay Standard Curve.

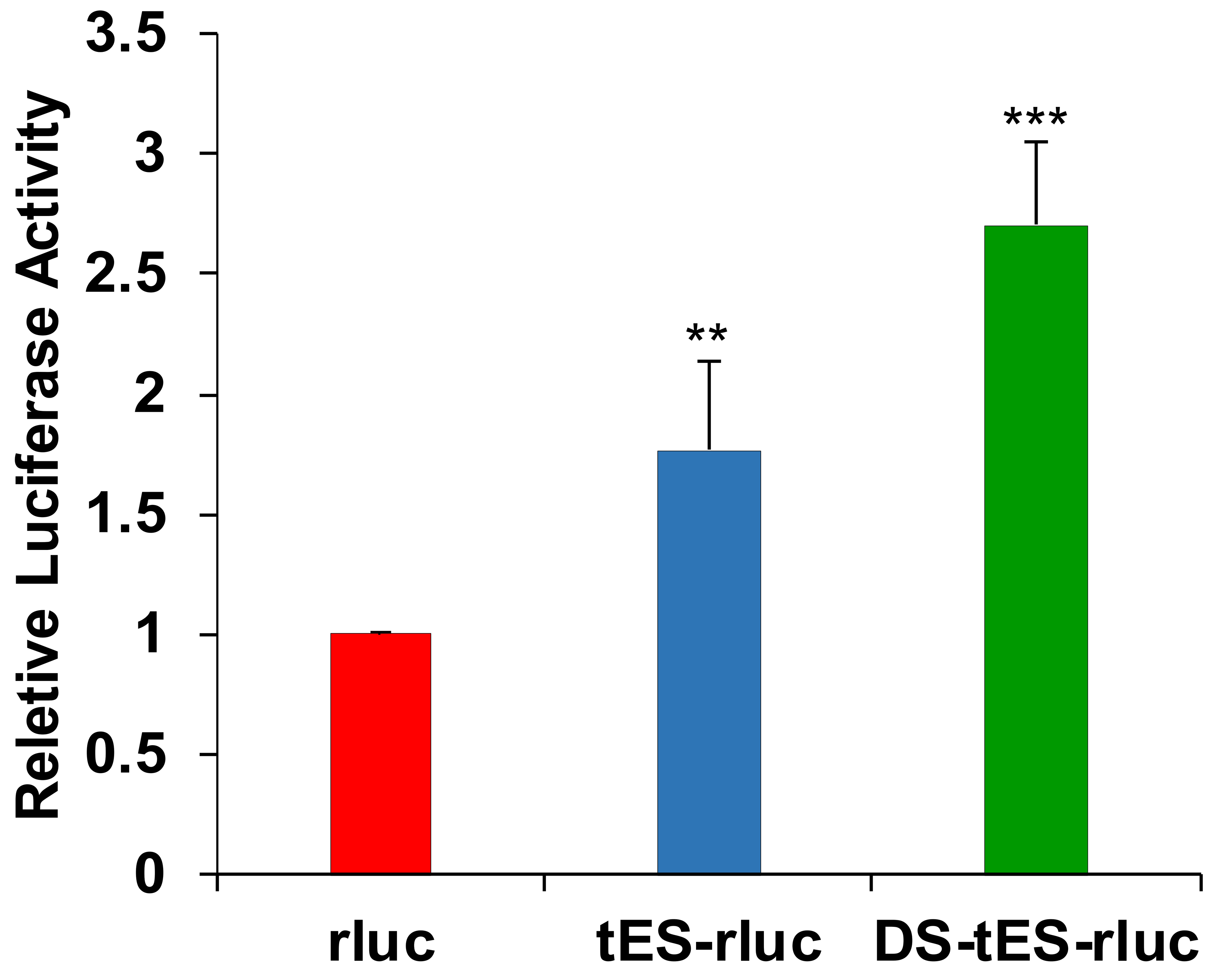

Figure S4. Permeability assay in Caco2 monolayers shows that both tES and DS-tES can permeabilize through intesti-nal epithelium. (Data are shown as mean ± SEM, n = 3. *** $P < 0.001$. and ** $P < 0.01$)

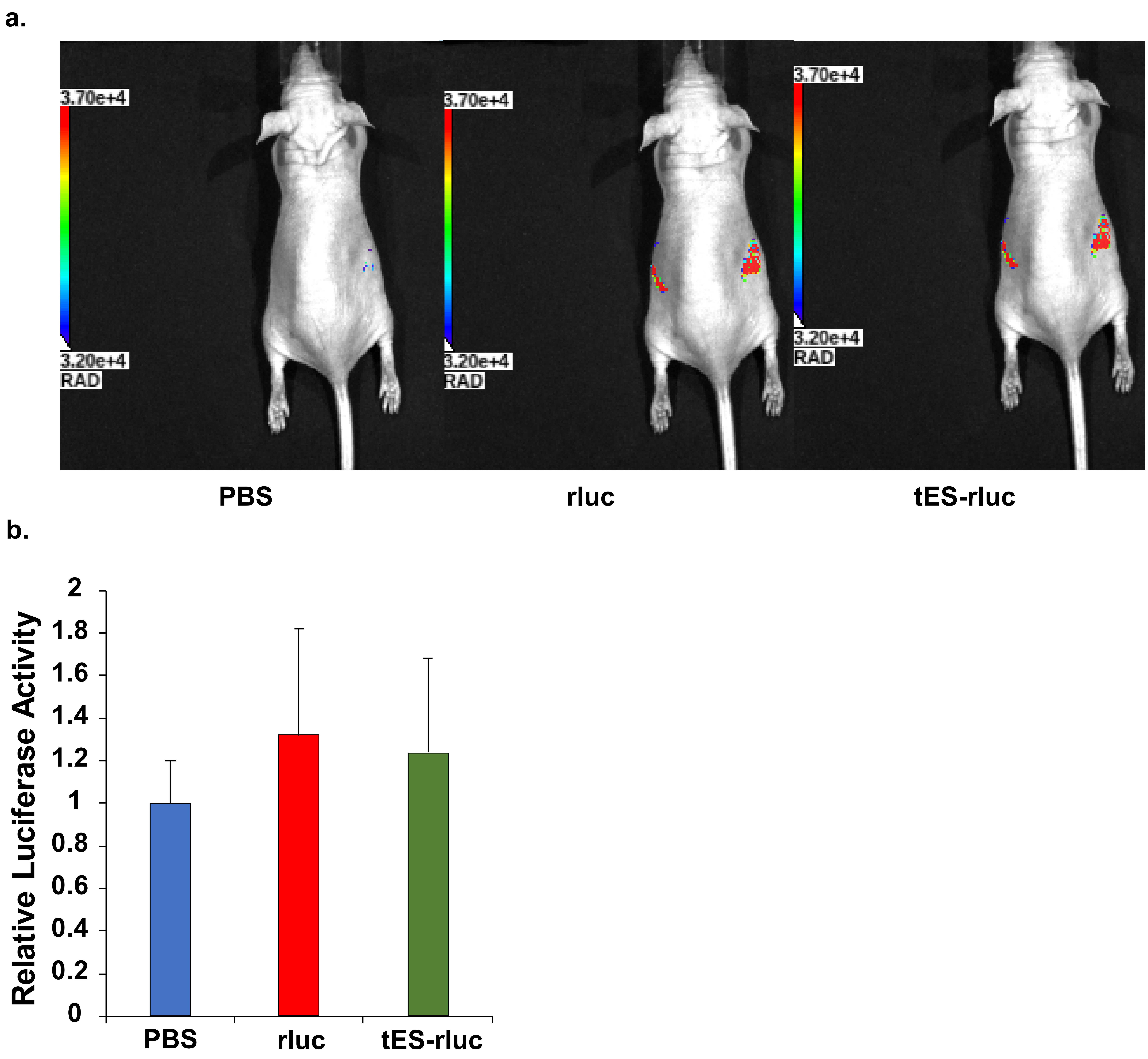

Figure S5.(a,b) Bioluminescence emitted from mice administered with tES-rluc/ rluc in 3 h.

**Features**	**DS-tES**	**tES**
**Number of cysteine per subunit**	5	0
**Non-reducing SDS-PAGE**	Majorly oxidized Shell	Subunits
**Molecular diameter**	~13 nm	~13 nm
**Stable at acidic pH (~4)**	Yes	No
**Against pepsin digestion at acidic pH**	Highly stable	Not stable
**Permeable through Caco2 monolayer**	High	High

Table S1. Comparative analysis of DS-tES and tES
